# Rabies research in Ethiopia: A systematic review

**DOI:** 10.1016/j.onehlt.2022.100450

**Published:** 2022-10-18

**Authors:** Aga E. Gelgie, Lisa Cavalerie, Mirgissa Kaba, Daniel Asrat, Siobhan M. Mor

**Affiliations:** a College of Veterinary Medicine and Agriculture, Addis Ababa University, P.O. Box 34, Bishoftu, Ethiopia; b Department of Animal Science, Institute of Agriculture, The University of Tennessee, 2506 River Drive Brehm Animal Science Bldg, TN, USA; c Institute of Infection, Veterinary and Ecological Sciences, University of Liverpool, Leahurst Campus, Neston CH64 7TE, UK; d International Livestock Research Institute, P.O. Box 5689, Addis Ababa, Ethiopia; e Addis Ababa University, College of Health Sciences, Addis Ababa, Ethiopia

**Keywords:** Rabies, Ethiopia, Incidence, Health Knowledge, Attitudes, Practice, Medicine, Traditional, DALYs, Disability-adjusted life years, KAP, Knowledge, attitudes and practices, NTDs, Neglected tropical diseases, PEP, Post-exposure prophylaxis, PRISMA, Preferred reporting items for systematic reviews and meta-analyses, SARE, Stepwise approach towards rabies elimination, SNNPR, Southern Nations, Nationalities, and Peoples’ region

## Abstract

Rabies is an important zoonosis in Ethiopia, where lack of research is cited as a constraint to implementation of the national rabies control strategy. We conducted a systematic review of publications and theses on rabies in Ethiopia, to document research gaps and areas of knowledge saturation in relation to geographic and species focus, methods and findings. We also examined funding sources and extent of local researcher participation. After screening titles and abstracts, the full text of 119 publications was included in data extraction. More than 40% of publications involved data collection in one region (Oromia); no publications reported findings from Benishangul-Gumuz, Dire Dawa or Gambella. Dogs and wildlife (especially *Canis simensis*) were the focus of research in 45% and 24% publications, respectively. Descriptive epidemiology (*N* = 39 publications), ethno-medicine/−pharmacology (*N* = 17) and knowledge, attitude, and practice surveys (KAP, *N* = 15) were amongst the most common study designs, while studies involving economic methods (*N* = 3) and experimental epidemiology to test interventions (*N* = 3) were under-represented. Incidence surveys (*N* = 9) commonly used post-exposure prophylaxis administration in humans as a proxy for exposure without laboratory confirmation of the rabies status of the animal. KAP surveys tended to highlight reasonable levels of knowledge of rabies and poor practices, including overreliance on medicinal plants. International researchers were the first or last (senior) author on 42% and 58% of publications, respectively, most of which were funded by international organizations (45/72 publications reporting funding source). Based on this systematic review, we suggest more applied research is needed to address gaps in laboratory surveillance (including in humans, domestic and wild animals); identify effective ways to overcome socio-cultural and other barriers to accessing effective rabies treatments; inform best approaches to incentivizing mass dog vaccination programs; and generate local estimates of the cost-benefit and cost-effectiveness of different control strategies to improve financing and political buy-in for rabies control in Ethiopia.

## 1 Introduction

Rabies is estimated to claim 59,000 human lives globally each year with wider societal costs estimated at over 3.7 million disability-adjusted life years (DALYs) [1]. Children under the age of 15 years constitute 40% of global cases with most of these occurring in Asia and Africa [2]. This makes the impact of the disease particularly burdensome in developing countries, where premature death contributes to productivity losses of future generations. In addition, rabies causes major economic losses associated with death of livestock in these agriculture-dependent countries [3]. Losses due to livestock death are estimated to cost 512 million USD every year, with African countries experiencing the greatest losses [1].

Transmission of rabies to humans and other mammals most often occurs following the bite of a domestic dog which transmits the virus in its saliva [4]. From the bite site, the virus travels along the peripheral nerves to the spinal cord and brain, where it causes inflammation and clinical signs such as hyperactivity and hydrophobia (furious form) or paralysis and coma (paralytic form) [5]. The disease is almost always fatal after the onset of clinical signs. However, prevention of human rabies is possible with rapid intervention, including washing of the bite wound and immediate treatment with post-exposure prophylaxis (PEP) and rabies immunoglobulin [6]. Vaccination of the domestic dog reservoir is considered the most cost-effective control measure and has contributed to the elimination of rabies in most developed countries [7].

Ethiopia has the second highest number of rabies-related deaths on the African continent [1]. The disease has been ranked the top zoonotic disease priority in Ethiopia by a panel of experts from human, animal, and environmental health [8]. Due to poor surveillance systems, precise national estimates of the disease burden are lacking. Recent research which extrapolated district case numbers to the national level estimated that as many as 97,000 people require PEP annually, costing the healthcare system USD 2 million each year; meanwhile, 3000 humans die each year due to infection [9]. Based on these figures, we estimate that Ethiopia represents only 1% of the global population but has 5% of the global burden of deaths due to rabies. Dog vaccination is mainly practiced in urban areas where vaccine supply is adequate and storage facilities are available [10]. A recent study has reiterated the reduced accessibility and affordability of dog vaccines in Ethiopia which is a bottleneck for achieving 70% vaccination coverage in dogs [11]. Furthermore, in many countries, including Ethiopia, PEP is limited to the central health facilities and only sporadically available in remote areas due to financial and logistical challenges, such as limited budget and supply shortages [12].

Efforts are underway to mitigate the impact of rabies, including promoting multi-sectoral engagement through use of the stepwise approach towards rabies elimination (SARE) tool [13]. The SARE tool provides a framework for addressing dog-mediated rabies by guiding countries through various steps to address gaps in legislation, data collection and analysis, laboratory diagnosis, advocacy and information, prevention, and control as well as dog population management and stakeholder collaboration. Following an initial self-assessment using the SARE tool, Ethiopia was classified as being in the very early stages of rabies control (stage 0 out of 5) [13]. More recently, the country has achieved a score of 2 out of 5, reflecting the fact that a national rabies control strategy has been drafted and is in the process of being implemented [14].

Amongst neglected topical diseases (NTDs), rabies is considered one of the most under-represented in terms of research investment relative to the burden of disease in children. [15]. This includes in Ethiopia, where lack of research is cited as a constraint to the national rabies control strategy [16]. Although the disease is the top zoonotic disease priority according to policymakers [8], the gaps in research as well as the areas where knowledge saturation has been reached have not been clearly articulated. To address this, we undertook a detailed systematic review of rabies research in Ethiopia to guide future research investments on this priority disease. The specific aims of the study were to:

i) Identify geographical regions where rabies research has been conducted in Ethiopia;
ii) Identify the species of focus of rabies research in Ethiopia;
iii) Assess the burden of rabies in Ethiopia in different species;
iv) Identify the research methodologies employed in rabies research in Ethiopia; and
v) Identify the funding source and extent of engagement of local researchers in rabies research in Ethiopia.

## 2 Material and methods

This systematic review was conducted in accordance with the Preferred Reporting Items for Systematic Reviews and Meta-analyses (PRISMA) guidelines (see supplementary file S1 for PRISMA-P checklist).

### 2.1 Search strategy

Electronic databases including Web of Science, PubMed, Scopus, AGRICOLA, AGRIS, Open Access Theses and Dissertations, WorldCat and Addis Ababa University library repository (MSc and PhD dissertations; http://etd.aau.edu.et/) were searched using the terms ‘Rabies AND Ethiopia’. Database searches were conducted between 7 August 2020 and 9 September 2020, with no restrictions on date of publication or article type. Papers written in English and French language were included. Search results from the databases were exported to Zotero Desktop software (Version 5.0.89).

### 2.2 Screening and data extraction

Screening and data extraction was performed using the web-based, systematic review software, Covidence (Veritas Health Innovation Ltd.; Melbourne, VIC) using a two-stage process. First, following automatic removal of duplicates, the title and abstract of each publication was screened by two authors to assess relevance. Studies deemed to have a partial or total focus on rabies in Ethiopia, including studies undertaken on rabies virus strains from Ethiopia as well as ethno-botanical treatments against rabies, were included. Studies conducted by Ethiopian researchers overseas but which did not focus on Ethiopia and studies with tangential reference to rabies were excluded. Studies with unclear relevance were included to enable review of the full text.

In the second stage, the full text of each publication was retrieved and reviewed by two authors. Data were extracted on: location of data collection and laboratory analysis; methodology(ies) employed (using high-level categories as adapted from [17]); species under study (e.g. human, dog, livestock, wildlife); authors' country and institutional affiliation; and organization and country origin of funding (see supplementary file S2 for data extraction template). Papers/theses which contained no relevant primary data or summary statistics (e.g. narrative review with limited data on rabies in Ethiopia) or which duplicated data reported elsewhere (e.g. thesis chapter which was subsequently published) were excluded at this stage. Each publication was reviewed by two authors independently and conflicts in extracted data were resolved by a third author where indicated.

### 2.3 Data analysis

Results of data extraction were exported to and analyzed in Excel version 2202 (Microsoft). Quantitative data were summarized as counts and proportions of publications by: location of data collection and laboratory analysis; species of focus; methods employed; funding sources; and authors' country and institutional affiliation. Further, the number of studies by zone was mapped in ArcMap version 10.5.1 (Esri) while incidence data was visualized using R version 3.6.3 (R Core Team). Since all studies were conducted before official separation of the Sidama region from the Southern Nations, Nationalities, and Peoples' region (SNNPR) in mid-2020, data collected from Sidama region were included under the SNNPR region. Given differences in methods and case definitions across publications we did not seek to do a formal meta-analysis to obtain a pooled estimate of incidence. However, where point estimates were provided for different years or locations in the same publication, a pooled estimate of the cumulative incidence was manually calculated using the raw data in the paper. Finally, characteristics and key findings of studies reporting incidence or knowledge, attitudes, and practices (KAP) were summarized in tables.

## 3 Results

**Fig. 1:**
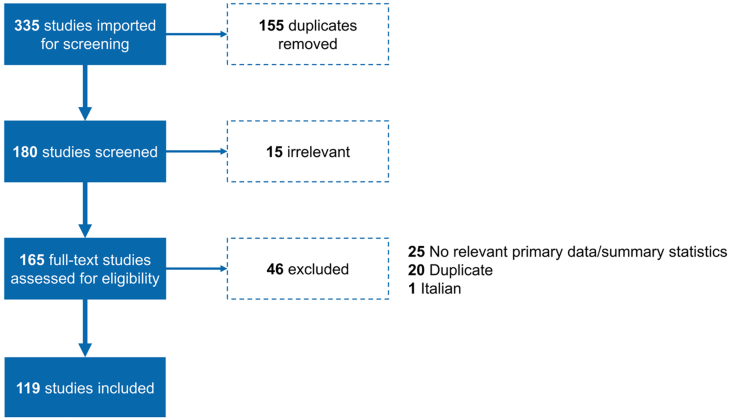
Preferred Reporting Items for Systematic Reviews and Meta-Analyses (PRISMA) flow diagram detailing number of papers on rabies in Ethiopia that were retrieved and selected for data extraction.

A total of 335 published papers and theses were imported for screening, of which 155 were excluded due to duplication. Subsequently, the titles and abstracts of 180 studies were screened for eligibility and a further 15 studies were excluded due to irrelevance (Fig. 1). The full text of 165 studies was assessed for eligibility with a further 46 studies excluded for the following reasons: no relevant primary data/summary statistics (*N* = 25); duplicate (*N* = 20); and text in Italian (*N* = 1). Thus, data extraction was performed on 119 studies, including 113 original research articles, 13 masters/PhD theses and 3 consultative meeting reports and conference proceedings. All studies included in data extraction were published between 1960 and 2020.

### 3.1 Geographic focus

**Fig. 2:**
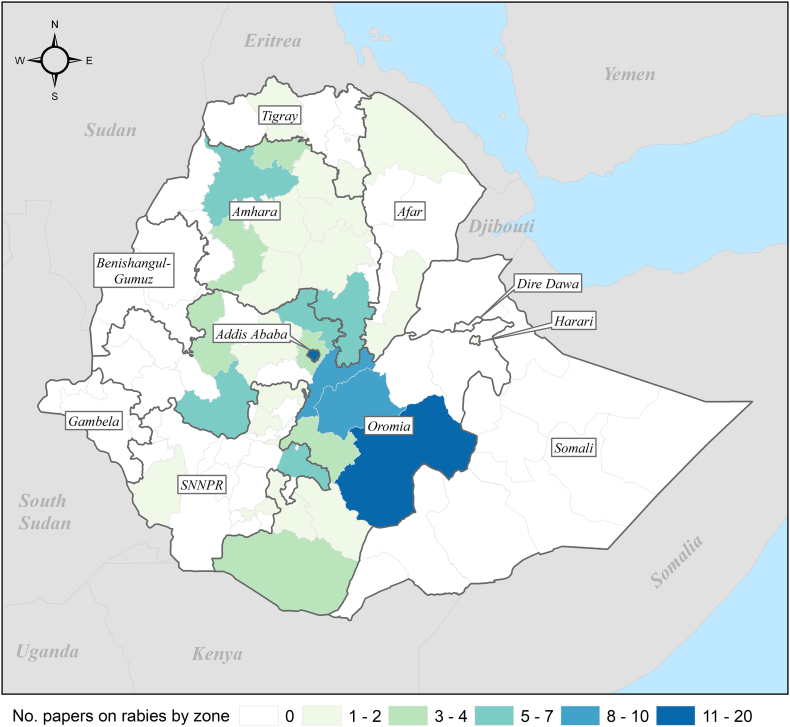
Map showing the number of publications on rabies in Ethiopia, by zone and region (N = 119). The names of regional states and chartered cities are indicated in boxes. SNNPR, Southern Nations, Nationalities and Peoples' Region.

Most of the studies reported on data collected exclusively in Ethiopia (*N* = 104, 87%) with remaining studies incorporating data from Ethiopia and other countries (United States, China, Kenya, and Sudan). Most studies involved data collection in Oromia regional state (*N* = 50, 42%), Addis Ababa (*N* = 20, 17%) and Amhara regional state (*N* = 17, 14%) (Table 1**;** Fig. 2). Only 7% (*N* = 9) of the studies focused on SNNPR, where approximately 20% of the Ethiopian population resides. No studies have been undertaken in Benishangul-Gumuz and Gambella regional states, nor in Dire Dawa city. Where laboratory analysis was performed (*N* = 37), this was undertaken in Ethiopia (*N* = 17, 46%) and overseas (*N* = 20, 54%).

**Table 1:** Number of publications on rabies in Ethiopia, by region (*N* = 119 publications). Since some papers focused on more than one region, the percentage does not add up to 100%.

Region/chartered city	No. of studies (%)	Human population in millions (%)^a^
Oromia	50 (42)	39.0 (38)
Addis Ababa	20 (17)	3.7 (4)
Amhara	17 (14)	22.5 (22)
SNNPR^b^	9 (8)	21.0 (20.5)
Tigray	6 (5)	5.6 (5.5)
Afar	3 (2.5)	1.9 (1.9)
Somali	1 (1)	6.3 (6)
Harari	1 (1)	0.3 (0.3)
Benishangul-Gumuz	0	1.2 (1.2)
Gambela	0	0.5 (0.5)
Dire Dawa	0	0.5 (0.5)
National	6 (5)	103.0 (100)
Not specified	1 (1)	–
Not applicable^c^	24 (20)	–

a Projected Population 2021, Central Statistical Agency of Ethiopia (CSA, 2021).

b Southern Nations, Nationalities, and Peoples' Region.

c No primary field data collection.

### 3.2 Species of focus

Most (69%) of the studies on rabies in Ethiopia focused on the animal domain (Table 2). Dogs received the greatest attention (45% of all studies). Studies of wildlife were also prominent (24% of all studies). These mostly focused on Ethiopian wolf (*Canis simensis*) populations in the Bale mountains (Oromia region) where there have been repeated rabies outbreaks and conservation efforts (*N* = 15). Only 3 studies report data on domestic dogs living in/around the Bale mountains.

**Table 2:** Number of publications on rabies in Ethiopia, by species of focus (N = 119 publications). Since some papers focused on more than one species, the percentage does not add up to 100%.

Species	No. of studies (%)
Human	67 (56)
Animal	82 (69)
Small animal	56 (47)
Dog	54 (45)
Cat	15 (13)
Livestock	24 (20)
Large ruminant	13 (11)
Small ruminant	5 (4)
Equine	11 (9)
Not specified^a^	9 (7.5)
Wildlife	28 (23.5)
Ethiopian wolf	18 (15)
Other wildlife^b^	21 (17.5)
Laboratory rodents^c^	20 (17)

a Mentioned in the studies only as ‘livestock’.

b Includes: antelope, monkey, leopard, fox, hyena, jackal, mongoose, African golden wolf, honey badger.

c Rat, rabbit, guinea pig, mice.

### 3.3 Research methods

**Fig. 3:**
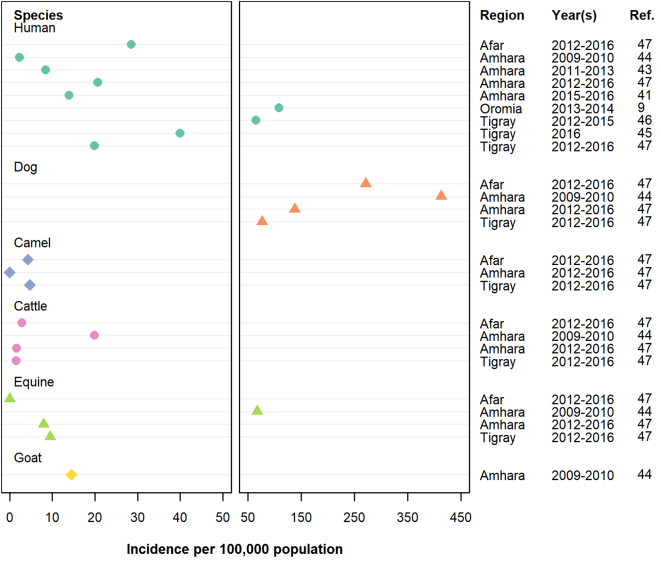
Cumulative incidence of rabies in Ethiopia, by species and region. Only those publications that reported incidence as cases per 100,000 are depicted (*N* = 7). Where possible, estimates from the same publication have been pooled to give a point estimate for each species and region. In all studies that reported incidence in humans and livestock, the rabies status of the dog that inflicted the bite was determined based on clinical criteria only (i.e. no laboratory confirmation).

The methodologies employed in rabies research are shown in Table 3. Most studies (N = 39, 33%) used descriptive epidemiology methods. In-vivo studies (N = 18, 15%), ethno-medicine/−pharmacology (N = 17, 14%), KAP surveys (N = 15, 13%) and laboratory studies (N = 14, 12%) were also relatively common, while observational epidemiology studies (testing risk factors; N = 4, 3.5%), experimental epidemiology studies (testing interventions; N = 3, 2.5%) and economic studies (N = 3, 2.5%) were under-represented. Of descriptive epidemiology studies, most reported on cases or outbreaks, while nine measured incidence (see supplementary file S3; Fig. 3). Case definitions used for incidence surveys were variable, although most used dog bite and consequent PEP administration as a proxy for rabies without laboratory confirmation of the dog. In-vivo studies – published from 1957 onwards – mainly focused on investigating rabies pathogenesis and evaluation of vaccine potency in laboratory rodents, while some studies examined the natural history of infection in the canine host. Ethno-medicine/−pharmacology studies – all but one of which was published in 2003 or later – investigated the use and effectiveness of specific plant species, such as *Phytolacca dodecandra* (commonly known as “endod”)*, Ricinus communis* (Castor bean or “qobo”) and *Nicotiana tabacum* (“tambo”), in the treatment of rabies*.* KAP surveys – all published in 2007 or later – were frequently used to assess pet management practices (such as confinement and vaccination of dogs) and health behaviours (such as wound management practices and use of medicinal plants/traditional healers to treat rabies) (see supplementary file S4). These studies tended to show reasonable levels of knowledge of rabies transmission but with attitudes and practices that were not always consistent with prevention. Finally, most of the studies involving laboratory methods focused on analysis of rabies outbreaks in the endangered Ethiopian wolves (*C. simensis*). The only intervention trials took place in the same setting.

**Table 3:** Number of publications on rabies in Ethiopia, by method (N = 119 publications). Since there are studies employing more than one method, the percentages do not add up to 100%.

Method	No. of studies (%)
Descriptive epidemiology (e.g. case reports/case series, incidence surveys)	39 (33)
In-vivo study (e.g. infection studies)	18 (15)
Ethno-medicine/−pharmacology	17 (14)
Knowledge, attitudes and practices (KAP) survey	15 (13)
Laboratory (e.g. genotyping of virus, production/testing vaccine in vitro)	14 (12)
Other social science study (e.g. qualitative interviews, focus group)	14 (12)
Ecology (e.g. dog behavior)	9 (7.5)
Narrative review/opinion/perspective	7 (6)
Participatory epidemiology (e.g. ranking, scoring, participatory mapping)	5 (4)
Mathematical or advanced statistical modelling (e.g. network analysis)	4 (3.5)
Observational epidemiology (e.g. cross-sectional, case-control, cohort)	4 (3.5)
Ecological/spatial modelling	3 (2.5)
Economics	3 (2.5)
Experimental epidemiology (e.g. clinical trial, intervention trial)	3 (2.5)
Systematic review/meta-analysis	2 (1.5)
Other^a^	17 (14)

a Includes: verbal autopsy (N = 2), policy (N = 3), diagnostic test evaluation (N = 2), evaluation of surveillance system (N = 1), letter (N = 1), online survey (N = 1), program evaluation (N = 1), meeting report (N = 1), secondary data analysis (N = 1), estimation of R0 (N = 1), questionnaire based on health belief model (N = 1), field observation with (N = 1) or without (N = 1) opportunistic sampling to determine cause of mortality.

### 3.4 Authorship and funding

Overall, almost half (*N* = 55, 46%) of the studies were conducted entirely by authors from Ethiopia, whereas around 40% (*N* = 47) were co-authored by researchers from Ethiopia and other countries (see supplementary file S5). Just over half (58%) of the papers had a first author from Ethiopia, while around one third of papers (29.5%) had a first author from the United Kingdom or United States. Similarly, around half the papers with multiple authors (50/97; 51.5%) had a last (senior) author from Ethiopia. Within Ethiopia, papers were published by researchers who were affiliated with Addis Ababa University (*N* = 21, 17.5%), Ethiopian Public Health Institute (*N* = 13, 11%; formerly known as Ethiopian Health and Nutrition Research Institute), Jimma University (*N* = 8, 6.5%), University of Gondar (*N* = 3, 2.5%) and Bule Hora University (N = 2, 1.5%).

Many of the studies (*N* = 47, 39.5%) did not state the sources of funds for the research. Where funding was stated, around one third of studies were fully (21/72; 29%) or partially (6/72; 8%) funded by organizations/institutions in Ethiopia. The remaining studies (*N* = 45; 62.5%) were funded exclusively by organizations/institutions from other countries. Of 16 studies on the Ethiopian wolf, 12 were funded by organizations/institutions from other countries.

## 4 Discussion

Although Ethiopia is on a mission to eliminate human rabies by 2030 [16], the current state of research in support of this goal has not been comprehensively analyzed. Here we provide a detailed, systematic literature review on rabies research undertaken in Ethiopia. We found significant gaps in geographic coverage of research with limited investment in applied research.

The lack of strong surveillance and relatively small number and geographic coverage of research studies on rabies makes it difficult to accurately assess the national burden of disease in Ethiopia. We found significant gaps in data, in particular in Benishangul-Gumuz and Gambella regional states, and Dire Dawa city. SNNPR was also under-studied relative to population size. Where incidence in humans was measured, dog bite was often used as a proxy without laboratory confirmation of the animal which inflicted the bite. Indeed, in 2018 etiological diagnosis of animal cases was only available at one laboratory, namely the Ethiopian Public Health Institute in the capital, Addis Ababa [16]. Given high rates of underreporting of rabies in Africa and elsewhere, bite injuries have been widely used to make predictions about the burden of human rabies [1,2,18]. However, the original methodology requires a series of adjustments to account for the probability that, for example, a dog which inflicts a bite injury may or may not be rabid, and a bite from a rabid dog may or may not result in rabies [18]. Most studies in Ethiopia instead equated the number of people who received PEP at a health center as a measure of incidence, irrespective of whether they were actually exposed and would have developed rabies. We also found rabies case definitions differed between publications making it difficult to compare incidence across regional states and amongst different animal species. Future research in animals would benefit from standardized case definitions.

Overall, we found most studies were largely descriptive and generated data that might be considered fairly low on the evidence pyramid [19]. In particular, KAP studies were mostly redundant in terms of information they generated, most of them pointing to poor practices albeit good knowledge. We found no publications to suggest that these studies were followed by interventions (research or otherwise) aimed at improving the identified poor practices. Amongst these poor practices, the use of medicinal plants that are not scientifically confirmed as effective rabies treatments has been widely researched in Ethiopia. A recent systematic review reported that rabies is the most common viral infection that is treated with medicinal plants in Ethiopia [24]. Rabies is one of few zoonotic infections where effective interventions – namely vaccination of dogs and PEP of humans – exist and elimination is considered within reach [25,26]. Therefore, we find calls by some researchers [24,27] for further investment into discovery of novel anti-rabies agents from plants is unwarranted. Since such practices delay on-time wound management and PEP administration at the health centers, research into, for example, barriers to reliably supplying health centers with and community access to PEP may prove a more worthwhile investment.

Since 99% of human rabies arise from dog bite [4], research into prevention strategies should revolve around canine rabies. While the high research investment directed to the Ethiopian wolf (15% of overall research investment in Ethiopia) is warranted given the endangered status of the animals [28] and associated conservation efforts, we note that the vast majority of this research was funded by international organizations and may not reflect the priorities of local institutions. Further, only three studies [[29], [30], [31]] have focused on dogs living adjacent wolf populations. It has been shown that the domestic dog population is essential for rabies persistence in wildlife ecosystems in other countries such as Tanzania [32], which again supports the need for more applied research investment on the domestic dog reservoir in Ethiopia. The recent study by Yoak and colleagues [11] – which identified door-to-door vaccination and subsidies as effective tools for overcoming barriers to dog vaccination in the capital, Addis Ababa – is an important study which could be expanded to rural areas, including those surrounding wildlife areas, where access to veterinary services is even more challenging.

We take this opportunity to call for more applied rabies research in Ethiopia to convince policymakers of the need for intervention and ensure political and community commitment toward the goal of rabies elimination. Targeted implementation research in Tanzania for example found a significant reduction in canine rabies incidence following mass vaccination program (70% after first campaign, 97% after second campaign) which also resulted in significant decline in need for PEP [33]. Similarly, in Chad, the cost-efficiency of PEP alone and PEP plus dog vaccination was compared and showed that dog vaccination was financially the best approach to control human rabies [34]. Research in that country also found that subsidizing dog vaccinations resulted in greater motivation for dog owners to vaccinate their dogs and to ultimately achieve the 70% dog vaccination milestone [11,35,36]. Finally, in Malawi, a classroom-based, educational intervention targeting primary school-aged children was found to be more effective in improving knowledge on rabies and its prevention than exposure to vaccine campaigns alone [37].

There are several limitations to this study. Although we attempted to include theses through searches conducted in relevant databases (i.e. Open Access Theses and Dissertations and Addis Ababa University library repository) it is possible we overlooked research conducted as part of degree studies if it did not culminate in publication and indexing in the peer-reviewed literature. Given the focus on research, we did not systematically consider surveillance reports or other grey literature except to provide context for the work. Owing to the variable way in which studies measured rabies incidence in humans and animals, we did not undertake a meta-analysis, although we note that one study did include a meta-analysis in their recent systematic review on rabies burden in Ethiopia [38]. Finally, following completion of our review we found a study reporting retrospective data on laboratory confirmed animal rabies cases in Addis Ababa and surrounds (2003–2009) [10] as well as two reporting rabies knowledge, attitudes and practices in Amhara [39,40]. Regrettably these publications were not included in our review, possibly because they were not indexed at the time our literature search ended in September 2020.

In conclusion, research into rabies in Ethiopia is inadequate considering the long-standing impact of the disease on human and animal health in the country. In order to achieve the goal of eliminating all human rabies deaths by 2030, more applied research is needed to: address gaps in laboratory surveillance (including in humans, domestic and wild animals); identify effective ways to overcome socio-cultural and other barriers to accessing effective rabies treatments; inform best approaches to incentivizing mass dog vaccination programs in urban and rural settings; and generate local estimates of the cost-benefit and cost-effectiveness of different control strategies to improve financing and political buy-in. Such applied research can aid and inform the implementation of the national rabies control and elimination strategy in Ethiopia.

## Funding

This work was supported by the Global Challenges Research Fund (GCRF) One Health Regional Network for the Horn of Africa (HORN) Project, from 10.13039/100014013UK Research and Innovation (UKRI) and 10.13039/501100000268Biotechnology and Biological Sciences Research Council (BBSRC) (project number BB/P027954/1); and the Soulsby Foundation (https://soulsbyfoundation.org/).

## CRediT authorship contribution statement

**Aga E. Gelgie:** Conceptualization, Data curation, Formal analysis, Investigation, Methodology, Project administration, Validation, Visualization, Writing – original draft, Writing – review & editing. **Lisa Cavalerie:** Conceptualization, Data curation, Formal analysis, Investigation, Methodology, Validation, Visualization, Writing – review & editing. **Mirgissa Kaba:** Conceptualization, Data curation, Formal analysis, Investigation, Validation, Writing – review & editing. **Daniel Asrat:** Conceptualization, Data curation, Formal analysis, Investigation, Validation, Writing – review & editing. **Siobhan M. Mor:** Conceptualization, Data curation, Formal analysis, Investigation, Methodology, Validation, Visualization, Writing – review & editing.

## Declaration of Competing Interest

None.

## Data Availability

Data will be made available on request.

## Appendix A Supplementary data

S1 PRISMA protocol Image 1

S2 data extraction template Image 2

S3 incidence studies Image 3

S4 KAP studies Image 3

S5 country affiliation of authors Image 3
