# Supplementary material for: Rabies research in Ethiopia: A systematic review
Source: One Health. 2022 Oct 18;15:100450. doi: 10.1016/j.onehlt.2022.100450 (PMC9754932; doi:10.1016/j.onehlt.2022.100450)
Supplement: Supplementary file 1 — S1 PRISMA protocol [file mmc1.docx]

**Supplementary file S1.** PRISMA-P 2015 checklist: recommended items to include in a systematic review PROTOCOL

| Section/topic | Item No. | Checklist item |
| --- | --- | --- |
| ADMINISTRATIVE INFORMATION | | |
| Title |  |  |
| Identification | 1a | *Identify the report as a protocol of a systematic review*  Rabies in Ethiopia: a systematic review |
| Update | 1b | *If the protocol is for an update of a previous systematic review, identify as such*  NA |
| Registration | 2 | *If registered, provide the name of the registry (e.g., PROSPERO) and registration number*  NA |
| Authors |  |  |
| Contact | 3a | *Provide name, institutional affiliation, and e-mail address of all protocol authors; provide physical mailing address of corresponding author*  Aga E. Gelgie^a,b^ [aga.edema@aau.edu.et](mailto:aga.edema@aau.edu.et)  Lisa Cavalerie^c,d^ [lisa.cavalerie@liverpool.ac.uk](mailto:lisa.cavalerie@liverpool.ac.uk)  Mirgissa Kaba^e^ [mirgissa.kaba@aau.edu.et](mailto:mirgissa.kaba@aau.edu.et)  Daniel Asrat^e^ [daniel.asrat@aau.edu.et](mailto:daniel.asrat@aau.edu.et)  Siobhan M. Mor^c,d*^ [siobhan.mor@liverpool.ac.uk](mailto:siobhan.mor@liverpool.ac.uk)  ^a^ College of Veterinary Medicine and Agriculture, Addis Ababa University, P.O.Box 34, Bishoftu, Ethiopia  ^b^ Department of Animal Science, Institute of Agriculture, The University of Tennessee, 2506 River Drive Brehm Animal Science Bldg, TN, USA  ^c^ Institute of Infection, Veterinary and Ecological Sciences, University of Liverpool, Leahurst Campus, Neston CH64 7TE, UK  ^d^ International Livestock Research Institute, P.O. Box 5689, Addis Ababa, Ethiopia  ^e^ Addis Ababa University, College of Health Sciences, Addis Ababa, Ethiopia  ^*^Corresponding author: Siobhan M. Mor. Email: [siobhan.mor@liverpool.ac.uk](mailto:siobhan.mor@liverpool.ac.uk) Postal address: Institute of Infection, Veterinary and Ecological Sciences, University of Liverpool, Leahurst Campus, Neston CH64 7TE, UK |
| Contributions | 3b | *Describe contributions of protocol authors and identify the guarantor of the review*  **Aga E. Gelgie** is the guarantor.  **Aga E. Gelgie:** Conceptualization, Data curation, Formal analysis, Investigation, Methodology, Project administration, Validation, Visualization, Writing – original draft, Writing – review & editing. **Lisa Cavalerie:** Conceptualization, Data curation, Formal analysis, Investigation, Methodology, Validation, Visualization, Writing – review & editing.  **Mirgissa Kaba:** Conceptualization, Data curation, Formal analysis, Investigation, Validation, Writing – review & editing.  **Daniel Asrat:** Conceptualization, Data curation, Formal analysis, Investigation, Validation, Writing – review & editing.  **Siobhan M. Mor:** Conceptualization, Data curation, Formal analysis, Investigation, Methodology, Validation, Visualization, Writing – review & editing. |
| Amendments | 4 | *If the protocol represents an amendment of a previously completed or published protocol, identify as such and list changes; otherwise, state plan for documenting important protocol amendments*  NA |
| Support |  |  |
| Sources | 5a | *Indicate sources of financial or other support for the review*  This work was supported by the Global Challenges Research Fund (GCRF) One Health Regional Network for the Horn of Africa (HORN) Project, from UK Research and Innovation (UKRI) and Biotechnology and Biological Sciences Research Council (BBSRC) (project number BB/P027954/1); and the Soulsby Foundation (https://soulsbyfoundation.org/).) |
| Sponsor | 5b | *Provide name for the review funder and/or sponsor*  NA |
| Role of sponsor/funder | 5c | *Describe roles of funder(s), sponsor(s), and/or institution(s), if any, in developing the protocol*  The funder played no role in the conceptualization or analysis of data for this systematic review. |
| INTRODUCTION | | |
| Rationale | 6 | *Describe the rationale for the review in the context of what is already known*   - Ethiopia has the second highest number of rabies-related deaths on the African continent - Efforts are underway to mitigate the impact of rabies, including promoting multi-sectoral engagement through use of the stepwise approach towards rabies elimination - Lack of research is cited as a constraint to the national rabies control strategy - Although the disease is the top zoonotic disease priority according to policy-makers, the gaps in research as well as the areas where knowledge saturation has been reached have not been clearly articulated |
| Objectives | 7 | *Provide an explicit statement of the question(s) the review will address with reference to participants, interventions, comparators, and outcomes (PICO)*  This review aims to:   1. Identify geographical regions where rabies research has been conducted in Ethiopia; 2. Identify the species of focus of rabies research in Ethiopia; 3. Assess the burden of rabies in Ethiopia in different species; 4. Identify the research methodologies employed in rabies research in Ethiopia; and 5. Identify the funding source and extent of engagement of local researchers in rabies research in Ethiopia. |
| METHODS | | |
| Eligibility criteria | 8 | *Specify the study characteristics (e.g., PICO, study design, setting, time frame) and report characteristics (e.g., years considered, language, publication status) to be used as criteria for eligibility for the review*  Study design: No restrictions  Setting: Studies deemed to have a partial or total focus on rabies in Ethiopia, including studies undertaken on rabies virus strains from Ethiopia as well as ethno-botanical treatments against rabies  Years: No restrictions  Language: English and French.  Publication status: published journal articles, theses (MSc, PhD) |
| Information sources | 9 | *Describe all intended information sources (e.g., electronic databases, contact with study authors, trial registers, or other grey literature sources) with planned dates of coverage*  Web of Science, PubMed, Scopus, AGRICOLA, AGRIS, Open Access Theses and Dissertations, WorldCat and Addis Ababa University library repository (MSc and PhD dissertations; <http://etd.aau.edu.et/>) |
| Search strategy | 10 | *Present draft of search strategy to be used for at least one electronic database, including planned limits, such that it could be repeated*  Rabies AND Ethiopia |
| Study records |  |  |
| Data management | 11a | *Describe the mechanism(s) that will be used to manage records and data throughout the review*  Search results from the databases will be exported to Zotero Desktop (Version 5.0.89) and imported into Covidence for screening and data extraction. |
| Selection process | 11b | *State the process that will be used for selecting studies (e.g., two independent reviewers) through each phase of the review (i.e., screening, eligibility, and inclusion in meta-analysis)*  Stage One: Two independent authors will screen title and abstract to assess relevance.  Stage Two: Two independent authors will review full text and complete data extraction template. |
| Data collection process | 11c | *Describe planned method of extracting data from reports (e.g., piloting forms, done independently, in duplicate), any processes for obtaining and confirming data from investigators*  Two independent authors will review full text and complete data extraction template in Covidence. Conflicting data will be resolved by a third author where indicated. |
| Data items | 12 | *List and define all variables for which data will be sought (e.g., PICO items, funding sources), any pre-planned data assumptions and simplifications*  Data will be extracted on: location of data collection and laboratory analysis; methodology(ies) employed, species under study (human, dog, livestock or wildlife), authors’ country and institutional affiliation; organization and country origin of funding |
| Outcomes and prioritization | 13 | *List and define all outcomes for which data will be sought, including prioritization of main and additional outcomes, with rationale*   - Region/zone - Species of focus - Research methods - Incidence studies: incidence, case definition, study design - KAP studies: indicators for knowledge, attitudes, practices - Ethnobotany studies: plant names - Authorship - Funding source |
| Risk of bias in individual studies | 14 | *Describe anticipated methods for assessing risk of bias of individual studies, including whether this will be done at the outcome or study level, or both; state how this information will be used in data synthesis*  NA |
| Data synthesis | 15a | *Describe criteria under which study data will be quantitatively synthesized*  NA – incidence taken directly from paper |
|  | 15b | *If data are appropriate for quantitative synthesis, describe planned summary measures, methods of handling data, and methods of combining data from studies, including any planned exploration of*  *consistency (e.g., I^2^, Kendall’s tau)*  NA |
|  | 15c | *Describe any proposed additional analyses (e.g., sensitivity or subgroup analyses, meta-regression)*  NA |
|  | 15d | *If quantitative synthesis is not appropriate, describe the type of summary planned*  NA |
| Meta-bias(es) | 16 | *Specify any planned assessment of meta-bias(es) (e.g., publication bias across studies, selective reporting within studies)*  NA |
| Confidence in cumulative evidence | 17 | *Describe how the strength of the body of evidence will be assessed (e.g., GRADE)*  NA |
