# Supplementary material for: Rabies research in Ethiopia: A systematic review
Source: One Health. 2022 Oct 18;15:100450. doi: 10.1016/j.onehlt.2022.100450 (PMC9754932; doi:10.1016/j.onehlt.2022.100450)
Supplement: Supplementary file 2 — S2 data extraction template [file mmc2.docx]

**Supplementary file S2.** Data extraction template for a systematic review of publications on rabies in Ethiopia

GENERIC INFORMATION

1) What is available for data extraction?

- Abstract only
- Abstract and full text

2) What is the type of publication?

- Published study
- Masters/PhD thesis
- Other ____________________

AUTHORSHIP AND FUNDING

3) What countries are the authors affiliated with?

*Consider primary (first listed) and secondary affiliations of ALL AUTHORS.*

- Ethiopia only
- Ethiopia and other countries
- Other countries only

4) If other countries, which countries?

________________________________________

5) Country affiliation of FIRST AUTHOR

*Consider PRIMARY (first listed) affiliation only.*

- Ethiopia
- Other ____________________

6) Institutional affiliation(s) of FIRST AUTHOR

________________________________________

7) Country affiliation of LAST AUTHOR.

*Consider PRIMARY (first listed) affiliation only.*

- Ethiopia
- Other ____________________

8) Institutional affiliation(s) of LAST AUTHOR

________________________________________

9) Country origin of funds

- Ethiopia
- Funding source not stated
- Other ____________________

10) Name of organisation(s) that funded the research

________________________________________

GEOGRAPHIC FOCUS

11) Country where data was collected

*If no data was collected (e.g. review, opinion) state the country(-ies) that the paper reports information on*

- Ethiopia only
- Other/multiple

12) If other/multiple, which country(-ies)?

________________________________________

13) For studies in Ethiopia: location of data collection

________________________________________

14) Country of laboratory analysis (if applicable)

- Ethiopia only
- Other/multiple
- Not applicable

15) If other/multiple, which country(-ies)?

________________________________________

METHODS

16) Method

*Retrospective studies using dog bite registration data hospitals = descriptive epidemiology (incidence survey)*

- In-vivo (i.e. animal study)
- Laboratory (e.g. genotyping of virus, production/testing vaccine in vitro)
- Descriptive epidemiology (e.g. case reports/case series/incidence survey/outbreak report) - studies which describe cases by person or animal/place/time
- Observational epidemiology (i.e. cross-sectional, case-control, cohort) - studies which identify and quantify risk factors, test hypotheses
- Experimental epidemiology (e.g. clinical trial, intervention trial) - compares intervention in different populations
- Mathematical or advanced statistical modelling (e.g. SIR, network analysis))
- Ecological/spatial modelling
- Participatory epidemiology (e.g. ranking, scoring, participatory mapping)
- Ecology (e.g. dog behaviour)
- KAP survey (i.e. assess knowledge, attitudes, practices)
- Ethnomedicine/ethnopharmacology (i.e. traditional medicines)
- Other social science study (e.g. qualitative interviews, focus group)
- Economics (e.g. DALYs, cost-benefit analysis)
- Narrative review/opinion/perspective
- Systematic review/meta-analysis
- Other ____________________

17) Species focus of the study

*Select "human" if study is an incidence survey of rabies using dog bites as proxy; Select "multiple" if study is a KAP and assesses human knowledge and dog management practices/husbandry/demography*

- Human
- Dog
- Other domestic animal (e.g. livestock)
- Wildlife
- Multiple

18) If other domestic/wildlife/multiple, which species?

________________________________________

INCIDENCE SURVEYS

19) How was rabies incidence measured?

- Laboratory testing
- Dog bite
- Verbal autopsy
- Not applicable
- Other ____________________

20) What was the population at risk?

*Denominator for incidence calculation e.g. population of health centre catchment area.*

________________________________________

21) What was the reported incidence?

*State incidence in numbers per population. Ensure it is clear if measure is incidence of bites or deaths.*

________________________________________

KAP SURVEYS

22) What indicators of KNOWLEDGE were used?

*E.g. rabies transmission, symptoms.*

________________________________________

23) What indicators of ATTITUDES were used?

*E.g. stray dogs, traditional medicine/treatment.*

________________________________________

24) What indicators of PRACTICES were used?

*E.g. health seeking behaviour, wound management, dog management.*

________________________________________

ETHNOMEDICINE/ETHNOPHARMACOLOGY STUDIES

25) Plant names

*Name of plant(s) used to treat/prevent rabies.*

________________________________________

OTHER COMMENTS

26) Add any other important comments here,

________________________________________
