# Supplementary material for: Rabies research in Ethiopia: A systematic review
Source: One Health. 2022 Oct 18;15:100450. doi: 10.1016/j.onehlt.2022.100450 (PMC9754932; doi:10.1016/j.onehlt.2022.100450)
Supplement: Supplementary file 3 — S3 incidence studies [file mmc3.docx]

**Supplementary file S4.** Publications reporting rabies incidence in Ethiopia (N=9 publications). Incidence is cases per 100,000 unless otherwise stated. In all studies reporting incidence in humans and livestock, the rabies status of the dog that inflicted the bite was not determined.

| Region | District(s) [zone(s)] | Year(s) | Species | Incidence | No. cases | Pop. at risk | Study design | Case definition | Comment | Ref. |
| --- | --- | --- | --- | --- | --- | --- | --- | --- | --- | --- |
| Amhara | Bahir Dar town [West Gojam] | 2015 | Human | 6.5 | 423 | 6,527,516 | Retrospective analysis of hospital records | Person who received PEP at health facility following exposure^a^ to suspected rabid dog | Study conducted at Addis Alem Hospital. Catchment area includes Bahir Dar city and three other zones in northwest Amhara. | [41] |
|  |  | 2016 |  | 7.5 | 501 | 6,642,874 |  |  |  |  |
| Amhara | Bahir Dar town; Bahir Dar Zuria; Mecha; and Yilmana Densa [all West Gojam] | 2014-2015 | Cattle | 0.01^b^ | 2 | 440 | Prospective, community-based study | Calf that was bitten by suspected rabid dog and developed nervous signs, extended recumbency, complete loss of appetite and eventually died | Study conducted in randomly selected farms in urban and peri-urban areas of Bahir Dar milk-shed which spans four districts (all mixed crop-livestock farming system). | [42] |
| Amhara | Gondar town [Central Gondar] | 2011 | Human | 4.6 | 140 | 3,042,865 | Retrospective analysis of hospital records | Person who received PEP at health facility following exposure^a^ to suspected rabid dog | Study conducted at Gondar health centre. Catchment area includes all of North Gondar Zone of Amhara. | [43] |
|  |  | 2012 |  | 2.6 | 81 | 3,101,544 |  |  |  |  |
|  |  | 2013 |  | 1.3 | 40 | 3,161,655 |  |  |  |  |
| Amhara | Dabat  [North Gondar]; and Gondar town [Central Gondar] | 2009-2010 | Human | 2.3^c^ | 3 | 128,146 | Prospective, community-based study | *Suspected case:* humans and animals showing symptoms consistent with rabies ^d^; *Exposure (probable) case:* animals and humans that were exposed^a^ to a known rabies suspect case | Study conducted in two purposely selected districts, from which 16 urban and rural villages (“kebelles”) were randomly selected. | [44] |
|  |  |  | Dog | 412.8^c^ | 13 | 3,149 |  |  |  |  |
|  |  |  | Cattle | 19.9^c^ | 9 | 45,526 |  |  |  |  |
|  |  |  | Equine | 67.7^c^ | 7 | 10,343 |  |  |  |  |
|  |  |  | Goat | 14.5^c^ | 2 | 13,842 |  |  |  |  |

| Oromia | Bishoftu  [East Shewa] | 2013-2014 | Human | 135 | 189 | 140,000 | Retrospective analysis of hospital records and active case finding in community | Person who was bitten by suspected rabid dog and who i) visited health facility (registered) or ii) was identified following active case finding in community (unregistered) | Study conducted in three purposively selected districts (B: urban; L-b: rural highland/mixed crop-livestock system; Y: rural lowland/pastoral system). | [9] |
| --- | --- | --- | --- | --- | --- | --- | --- | --- | --- | --- |
|  | Limu Bilbilo (LB) [Arsi] |  |  | 101 | 189 | 187,000 |  |  |  |  |
|  | Yabelo [Borena] |  |  | 86 | 87 | 101,000 |  |  |  |  |
| Oromia | Dugda Dawa (DD) [West Guji] | 2013-2014 | Cattle | 2.2%^e^ | NS^f^ | 2,325 | Questionnaire with farmers | Cow that was bitten by suspected rabid dog and/or which showed clinical signs^g^ without known exposure in past year. | Study conducted in four purposely selected districts (M and LB: mixed crop-livestock system; Y and DD: pastoral system), from which 20 villages were randomly selected. | [3] |
|  | Limu Bilbilo (LB) [Arsi] |  |  | 2.4%^e^ | NS^f^ | 1,871 |  |  |  |  |
|  | Munessa (M) [Arsi] |  |  | 2.1%^e^ | NS^f^ | 1,345 |  |  |  |  |
|  | Yabelo (Y) [Borena zone] |  |  | 1.1%^e^ | NS^f^ | 2,171 |  |  |  |  |
| Tigray | Shire  [North Western] | 2016 | Human | 40 | 368 | 920,169 | Prospective, hospital-based study | Person who received PEP at health facility following exposure^a^ to suspected rabid dog | Study conducted at Suhul hospital. Catchment area includes eight districts in northeast Tigray. | [45] |
| Tigray | Shire  [North Western] | 2012 | Human | 35.8 | 288 | Not stated | Retrospective analysis of hospital records | Person who received PEP at health facility following exposure^a^ to suspected rabid dog | Study conducted at Suhul hospital. Catchment area includes eight districts in northeast Tigray. | [46] |
|  |  | 2013 |  | 63.0 | 515 |  |  |  |  |  |
|  |  | 2014 |  | 89.8 | 747 |  |  |  |  |  |
|  |  | 2015 |  | 73.1 | 630 |  |  |  |  |  |

*Continued on next page*

| Multiple [Amhara, Afar and Tigray] | Ab’Ala  [Zone 2, Afar] | 2012-  2016 | Human | 28.5 | 14 | 49,205 | Retrospective analysis of hospital and agriculture and animal health offices records (includes laboratory tests for dogs) | *Human*: Rabid dog bite cases were considered as rabies positive  *Dog*: Brain samples from rabid dogs were confirmed by histopathology and direct fluorescent antibody tests at the Ethiopian Public Health Research  Institute |  | [47] | |
| --- | --- | --- | --- | --- | --- | --- | --- | --- | --- | --- | --- |
|  |  |  | Dog | 271.0 | 2 | 738 |  |  |  |  |  |
|  |  |  | Camel | 4.3 | 1 | 23,069 |  |  |  |  |  |
|  |  |  | Cattle | 2.9 | 1 | 33,938 |  |  |  |  |  |
|  |  |  | Equine | 0.0 | 0 | 7,125 |  |  |  |  |  |
|  | Alamata [Southern zone, Tigray] |  | Human | 17.4 | 26 | 149,567 |  |  |  |  |  |
|  |  |  | Dog | 37.0 | 1 | 2,700 |  |  |  |  |  |
|  |  |  | Camel | 0.0 | 0 | 7,452 |  |  |  |  |  |
|  |  |  | Cattle | 1.6 | 2 | 123,519 |  |  |  |  |  |
|  |  |  | Equine | 6.8 | 1 | 14,793 |  |  |  |  |  |
|  | Endamehoni [Southern zone, Tigray] |  | Human | 13.7 | 18 | 131,560 |  |  |  |  |  |
|  | Guba Lafto and Woldiya  [North Wello, Amhara] |  | Human | 33.2 | 77 | 231,718 |  |  |  |  |  |
|  |  |  | Dog | 139.0 | 3 | 2,159 |  |  |  |  |  |
|  |  |  | Camel | 0.0 | 0 | 1,289 |  |  |  |  |  |
|  |  |  | Cattle | 4.2 | 4 | 96,316 |  |  |  |  |  |
|  |  |  | Equine | 10.0 | 2 | 19,965 |  |  |  |  |  |
|  | Ofla  [Southern zone, Tigray] |  | Human | 17.9 | 25 | 139,622 |  |  |  |  |  |
|  |  |  | Dog | 59.8 | 1 | 1,672 |  |  |  |  |  |
|  |  |  | Camel | 0 | 0 | 0 |  |  |  |  |  |
|  |  |  | Cattle | 1.4 | 2 | 138,775 |  |  |  |  |  |
|  |  |  | Equine | 11.2 | 2 | 17,923 |  |  |  |  |  |
|  | Raya Azebo [Southern zone, Tigray] |  | Human | 29.1 | 47 | 161,566 |  |  |  |  |  |
|  |  |  | Dog | 139.0 | 3 | 2,159 |  |  |  |  |  |
|  |  |  | Camel | 7.4 | 1 | 13,589 |  |  |  |  |  |
|  |  |  | Cattle | 1.6 | 2 | 123,519 |  |  |  |  |  |
|  |  |  | Equine | 10.8 | 1 | 9,287 |  |  |  |  |  |
|  | Raya Kobo [North Wello, Amhara] |  | Human | 8.4 | 20 | 237,349 |  |  |  |  |  |
|  |  |  | Dog | 136.3 | 2 | 1,467 |  |  |  |  |  |
|  |  |  | Camel | 0.0 | 0 | 12,506 |  |  |  |  |  |
|  |  |  | Cattle | 0.5 | 1 | 213,515 |  |  |  |  |  |
|  |  |  | Equine | 5.7 | 1 | 17,473 |  |  |  |  |  |
| ^a^ Exposure defined according to existing WHO criteria, namely: nibbling of uncovered skin, minor scratches or abrasions without bleeding (exposure; Category II); single or multiple transdermal bites or scratches, contamination of mucous membrane or broken skin with saliva from animal licks, exposures due to direct contact with bats (severe exposure; Category III).  ^b^ Incidence rate expressed as deaths per calf-month at risk, i.e. denominator in incidence calculation reflects actual period of follow up of calves adjusted for loss-to-follow up.  ^c^ Incidence calculated as sum of suspect and probable cases.  ^d^ Signs consistent with rabies included: encephalitis with spasm in response to sensory stimuli, change of temper, vocalization, drooling, paralysis and other neurological signs  ^e^ Incidence calculated at herd-level and individual-level for each village. Only individual-level incidence is reported here.  ^f^ Seventy-two cases across all four districts. Number of cases by district was not stated (NS).  ^g^ Clinical signs described by the owner were triangulated with descriptions of rabies in cattle in the scientific literature. Owner statements about cattle that were bitten and/or were sick with rabies were also verified by the neighbor. | | | | | | | | | | |  |
