# Supplementary material for: Rabies research in Ethiopia: A systematic review
Source: One Health. 2022 Oct 18;15:100450. doi: 10.1016/j.onehlt.2022.100450 (PMC9754932; doi:10.1016/j.onehlt.2022.100450)
Supplement: Supplementary file 4 — S4 KAP studies [file mmc4.docx]

**Supplementary file S5.** Publications reporting knowledge, attitudes and practices related to rabies in Ethiopia (N=15). Percentages indicate the proportion of respondents who had that knowledge/attitude/practice.

| Region | District(s) [zone(s)] | Year(s) | Participants | Sample size | Knowledge | Attitude | Practices | Ref. |
| --- | --- | --- | --- | --- | --- | --- | --- | --- |
| Addis Ababa | Addis Ketema, Akaki-Kalit, Arada, Bole, Kolfe-Keran, Yeka [Region 14] | 2003 | Urban residents (random sample) | 2,390 | Know dogs/cats/ other animals transmit rabies: 97.2%  Know rabies is transmitted by bite/scratch/lick: 73.4%  Know wound management and PEP prevents rabies in humans: 85.9% | Believe PEP and traditional medicine prevents rabies: 12.1% | Owns dog: 40.5%  Confines dog: 47.8% Vaccinates dog: 33.3% | [48] |
| Addis Ababa | All sub-cities [Region 14] | 2011 | Household heads (random sample) | 1,260 | Heard of rabies before: 83%  Know rabies affects all warm blooded animals, with dogs the major vector: 73.5%  Know bites are the main mode of transmission to humans: 75.6%  Recognize clinical signs: 34%  Known vaccination prevents rabies in animals: 46.6%  Know preventive measures are available for humans: 28.7% | Willing to vaccinate pets: 98%  Believe it is important to seek medical attention after dog bite: 98.2%  Believe traditional medicine can prevent/treat rabies: 58.3%  Support mass dog vaccination and euthanasia of stray dogs: 95% | Owns dog: 25.3%  Confines dog: 89.5% Vaccinates dog: 43.6% | [49] |
| Addis Ababa | Arada, Gulele, Kirkos, Yeka [Region 14] | - | Dog owners  (random sample) | 252 | Know rabies is transmitted from dog to human: 95.4% | - | - | [50] |
| Amhara | Gondar city [Central Gondar], Dabat [North Gondar] | 2009-2010 | Dog owners (random sample) | 120 | Heard of rabies before: 98%  Know bite is main mode of transmission: 98% | - | Vaccinates dog: 20% Use traditional medicine after bite: 84% | [44] |

*Continued on next page*

| Amhara | Gondar Zuria [Central Gondar] | 2013 | Household heads (random sample) | 400 | Heard of rabies before: 99.3%  Know dog is most common source: 99.3%  Know bite is main mode of transmission: 94%  Know aggression is sign in animals: 63.5% | Believe rabies is important problem: 92.3%  Believe rabies is fatal once signs develop: 67.8%  Believe consumption of cooked/boiled meat from rabid animals is safe: 67.0%  Believe consumption of raw meat from rabid animals is safe: 19.0%  Prefer modern medicine: 38.8%  Prefer traditional medicine: 35.0% | Wash wound with water and soap after bite: 30.7%  Seek medical care after bite: 12.1%  Use traditional medicine after bite: 31.9% | [21] |
| --- | --- | --- | --- | --- | --- | --- | --- | --- |
| Amhara | Dera, Ebenat, Debre Tabor [South Gondar] | 2017 | Household members (random sample) | 384 | Heard of rabies before: 77.9%  Know rabies is transmitted from animal to human: 56%  Know bite is main mode of transmission: 71.1%  Know change of behavior is sign in animals: 75.8%  Know rabies is fatal once signs develop: 84.6%  Know about PEP: 39.6%  Know rabies can be prevented with vaccination: 65.9% | Willing to vaccinate pets: 69.8%  Believe traditional medicine can cure rabies: 81.5%  Believe consuming meat from rabid animals can cure rabies: 48.2%  Believe burning rabid animals and inhaling smoke can cure rabies: 0%  Believe that crossing a river within 40 days prevents disease development: 10.9% | Owns dog: 42.4%  Confines dog: 40.1%  Vaccinates dog: 19.8%  Wash wound with soap and water after bite: 8.9%  Seek medical care after bite: 30.5%  Use traditional medicine after bite: 58.7% | [20] |
| Oromia | Mena, Limu Kosa [Jimma] | 2011-2012 | Dairy farmers (random and purposive sample) | 294 | Heard of rabies before: 83.3%  Know rabies is transmitted from animals: 94.7%  Know means of transmission: 92.5% | - | Owns dog: 29.6%  Confines dog: 44.8%  Vaccinates dog: 4.6% | [51] |
| Oromia | Bishoftu town [East Shewa] | 2016 | Dog owners (random sample) | 249 | - | Strong intention to vaccinate dogs: 60% | Vaccinates dog: 38% | [52] |

*Continued on next page*

| Oromia | Goma [Jimma] | 2014-2015 | Human and animal health service providers (all) | 351 | Heard of rabies before: 99.7%  Know rabies is transmitted from animals to humans: 98.1%  Know that vaccination prevents rabies in animals: : 73.9% | - | - | [53] |
| --- | --- | --- | --- | --- | --- | --- | --- | --- |
| Oromia | Jimma town [Jimma] | 2012-2013 | Animal bite victims (purposive sample) | 384 | Heard of rabies before: 91.7%  Know rabies is transmitted by bite/lick of rabid dog: 99.0%  Know that vaccination prevents rabies in animals: 41.7% | Believe individuals should seek medical attention after dog bite: 91.1%  Believe traditional healers can cure rabies: 75.8%  Believe rabies is preventable: 75%  Believe free roaming dogs are risky: 92.7% | Owns dog: 48.4%  Confines dog: 8.6%  Vaccinates dog: 4.8%  Wash wound with soap and water after bite: 7.0% | [23] |
| Oromia | Sibu Sire [East Wellega] | - | Teachers (T), students (S), farmers (F), nurses (N) (stratified random sample) | 428; 13 (T), 79 (S), 299 (F), 37 (N) | Know signs and mode of transmission: 54% (T), 45% (S), 43% (F), 100% (N) | - | - | [54] |
| Tigray | Ayder, Kedamay Weyane [Mekelle special] | 2016 | Household heads (random sample) | 633 | Heard of rabies before: 87.8%  Know rabies affects all warm blooded animals, including humans: 74.2%  Know rabies is prevented by vaccination: 69.8%  Know rabies is treated by PEP: 67.6%  Know rabies is fatal disease: 45.7% | Believe stray dogs are dangerous: 82.9%  Willing to register pets: 56.9%  Believe holy water cures rabies: 49.8% | Have contact with dog/cat: 60.5%  Confines dog: 96.7%  Vaccinates dog: 79.3%  Seek medical care after bite: 77.5%  Seek traditional healer after bite: 10.1%  Use holy water after bite: 12.3% | [22] |

*Continued on next page*

| Multiple [Oromia and Tigray] | Asela Town (A)  [Arsi, Oromia], Mekelle special (M)  [Tigray] | 2014-2015 | Dog owners, community members, municipality workers, and veterinary and medical professionals  (purposive sample) | 398;  146 (A), 252 (M) | Heard of rabies before: 90.4% (A), 94.8% (M)  Know rabies is transmitted by animals: 73.5% (A), 89.5% (M)  Know route of transmission: 81.1% (A), 79.5% (M)  Know dogs are main animal affected: 98.4 % (A), 95.4% (M) | Believe rabies can be controlled by eradicating stray dogs: 6.1% (A), 33.9% (M) | Owns dog/cat: 48.6% (A), 94.8% (M)  Confines dog: 83.7% (A), 68.6 (M)  Vaccinates dog: 28.8% (A), 43.1% (M)  Wash wound with soap and water after bite: 39.7% (A), 22.6% (M)  Use traditional medicine after bite: 13.0% (A), 38.5% (M) | [55] |
| --- | --- | --- | --- | --- | --- | --- | --- | --- |
| Multiple [Afar and Oromia] | Metehara Town [East Shewa, Oromia], Merti [Arsi, Oromia] and surrounds | 2012 | Urban residents (U), pastoralists (P), health workers (H) (all) | 539; 471 (U), 49 (P), 19 (H) | Heard of rabies before: 98.5% (U), 91.8% (P); 100% (H)  Know rabies is transmitted to people by animal bite: 92% (U), 95.6% (P), 100% (H)  Know change of behavior is sign in dogs: 0% (U), 4.2% (P), 37.5% (H)  Know aggression is sign in dogs: 10.8% (U), 18.8% (P), 75% (H)  Know rabies is fatal in humans: 52.4% (U), 20% (P), 100% (H) | Willing to pay for dog vaccination: 99% (U), 73.5% (P) | Owns dog: 33.3% (U); 75.5% (P)  Seek medical care after bite: 99% (U)  Seek traditional healer after bite: 1% (U), 58.7% (P) | [56] |
| Multiple [Addis Ababa, Oromia] | Addis Ababa; Holeta town, Sabeta, [Finefine Special, Oromia]; Bishoftu town [East Shewa, Oromia] | 2008-2009 | Elementary and high school students (S), graduate students (G), human and animal health professionals (H), and non-health professionals (N)(stratified random sample) | 576; 192 (S), 192 (G), 96 (H), 96 (N) | Heard of rabies before: 100% | - | - | [57] |
