# Supplementary material for: Rabies research in Ethiopia: A systematic review
Source: One Health. 2022 Oct 18;15:100450. doi: 10.1016/j.onehlt.2022.100450 (PMC9754932; doi:10.1016/j.onehlt.2022.100450)
Supplement: Supplementary file 5 — S5 country affiliation of authors [file mmc5.docx]

**Supplementary file S3.** Country affiliation of first and last (senior) authors of publications on rabies in Ethiopia (N=119 publications).

| **Country affiliation of author(s)** | | **Number of studies (%)** |
| --- | --- | --- |
| All authors on paper | Ethiopia only | 55 (46) |
|  | Ethiopia and other countries | 47 (39.5) |
|  | Other countries only | 17 (14) |
| First authors | Ethiopia | 69 (58) |
|  | United Kingdom | 22 (18.5) |
|  | United States | 13 (11) |
|  | The Netherlands | 5 (4) |
|  | South Africa | 2 (1.5) |
|  | Others^a^ | 6 (5) |
|  | Not stated | 2 (1.5) |
| Last authors | Ethiopia | 50 (42) |
|  | United Kingdom | 15 (12.5) |
|  | United States | 12 (10.5) |
|  | The Netherlands | 6 (5) |
|  | Germany | 3 (2.5) |
|  | Canada | 2 (1.5) |
|  | Others^b^ | 9 (7.5) |
|  | Not applicable (single author) | 22 (18.5) |
| ^a^ Germany, Italy, Malaysia, Norway, Spain, Sweden  ^b^ Australia, China, Ghana, India, South Africa, Spain, Sudan, United Arab Emirates | | |
